# Biogenic synthesis and characterization of selenium nanoparticles by halotolerant *Bacillus sonorensis* 2MNHR

**DOI:** 10.1007/s00253-026-13921-y

**Published:** 2026-08-15

**Authors:** Nourhan G. Naga, Mona E. M. Mabrouk, Radwa M. Taha, Mervat A. Arayes, Hend M. H. Al-Kordy

**Affiliations:** 1 Department of Botany and Microbiology, Faculty of Science, Alexandria University, Alexandria, Egypt; 2 Botany and Microbiology Department, Faculty of Science, Damanhour University, Damanhour, Egypt

**Keywords:** Selenium nanoparticles, Biogenic synthesis, Halotolerant marine *Bacillus sonorensis*, Selenite bioreduction, Physicochemical characterization, Antimicrobial activity

## Abstract

**Abstract:**

Selenium nanoparticles (SeNPs) have attracted significant attention owing to their unique physicochemical properties and promising biomedical applications. In the present study, a newly isolated halotolerant marine *Bacillus sonorensis* 2MNHR strain was employed for the extracellular biosynthesis of SeNPs. Bacterial isolates capable of reducing selenium oxyanions were obtained from seawater samples collected at Alexandria Harbor, Mediterranean Sea, Egypt. The ability of ten morphologically distinct bacterial isolates to reduce sodium selenite and sodium selenate under aerobic conditions was evaluated. Among them, only one isolate consistently reduced sodium selenite, as evidenced by the development of a characteristic red coloration in both liquid and solid media within 72 h, whereas no reduction was observed with sodium selenate. Extracellular reduction was confirmed using a cell-free supernatant assay. UV-Vis and FTIR analyses of the filtrate confirmed the involvement of biomolecules in selenite biotransformation, while GC–MS analysis revealed diverse organic compounds potentially responsible for reduction and stabilization. The biosynthesized SeNPs were comprehensively characterized using UV-visible spectroscopy, Fourier-transform infrared spectroscopy (FTIR), X-ray diffraction (XRD), zeta potential analysis, scanning electron microscopy coupled with energy-dispersive X-ray spectroscopy (SEM–EDX), and transmission electron microscopy (TEM). These analyses confirmed the formation of spherical, amorphous nanoparticles with sizes ranging from 80 to 200 nm (average ~ 160 nm) and a characteristic UV-Vis peak at 226 nm. A zeta potential of −38 mV indicated high colloidal stability. The SeNPs exhibited strong antimicrobial activity, with inhibition zones ranging from 23 to 32 mm and MIC values from 19.5 to 156.25 µg/mL. MBC/MIC ratios (≤ 2) confirmed bactericidal activity against most tested bacteria, while a ratio of 4 indicated borderline activity against *Pseudomonas aeruginosa* and fungistatic behavior against *Candida albicans*. These findings highlight the potential of halotolerant marine bacteria as sustainable platforms for the production of stable and biologically active SeNPs.

**Key points:**

• *Isolation of marine Bacillus sonorensis 2MNHR with high selenite-reducing potential*

• *Efficient extracellular biosynthesis of stable selenium nanoparticles (SeNPs)*

• *Integrated physicochemical characterization and antimicrobial evaluation of SeNPs*

## Introduction

Selenium (Se) is an essential trace element incorporated into more than 25 selenoproteins, including glutathione peroxidase and thioredoxin reductase, which are crucial for maintaining cellular redox homeostasis (Xiao et al. 2023; Alsafran et al. 2025). In natural environments, selenium occurs in multiple oxidation states, primarily as selenate (SeO₄^2^⁻), selenite (SeO₃^2^⁻), and elemental selenium (Se^0^). While selenium is essential at trace concentrations, elevated concentrations, particularly of selenite, are highly toxic, prompting the development of microbial detoxification strategies in contaminated ecosystems (Eswayah et al. 2016). Conversely, selenium deficiency has been associated with disorders such as Keshan disease and impaired immune function, highlighting the narrow margin between nutritional requirement and toxicity (Shirazi et al. 2025). Selenium nanoparticles (SeNPs) have emerged as promising nanomaterials due to their distinct physicochemical properties, including particle sizes typically ranging from 50 to 400 nm, spherical morphology, structural stability, and enhanced bioavailability compared to bulk selenium (Huang et al. 2023; Xiao et al. 2023). These features support their potential applications in antimicrobial, anticancer, and antioxidant therapies, offering comparatively lower toxicity and improved bioactivity. Notably, biogenic SeNPs have demonstrated significant antimicrobial activity against Gram-negative bacteria, such as *Pseudomonas aeruginosa* and *Escherichia coli*, often exceeding that of their chemically synthesized counterparts through mechanisms involving membrane disruption and the generation of reactive oxygen species (ROS) (Alsafran et al. 2025; Shirazi et al. 2025). In the context of increasing antimicrobial resistance, recent studies have further highlighted their potential as alternative antimicrobial agents (Ansari et al. 2024). Regarding the biological basis of SeNP formation, microorganisms contribute substantially to the selenium biogeochemical cycling system by reducing soluble selenium oxyanions into insoluble elemental Se^0^ via assimilatory or dissimilatory pathways (Eswayah et al. 2016; Wang et al. 2022). Selenite reduction generally occurs more readily and may involve nitrate reductases or glutathione-mediated systems, leading to the formation of stable elemental selenium deposits; in contrast, selenate reduction requires additional respiratory processes and is typically less efficient (He and Yao 2010). This microbial transformation not only mitigates selenium toxicity but also enables the biosynthesis of functional nanomaterials naturally capped with biomolecules that enhance nanoparticle stability and biological activity (Eswayah et al. 2016). Compared with chemical and physical synthesis methods, which often require hazardous stabilizers and high energy inputs, bacterial-mediated biosynthesis offers an eco-friendly and cost-effective alternative (Mikhailova 2023; Alsafran et al. 2025). Both intracellular and extracellular biosynthetic routes have been reported, involving enzymatic reduction within cells or the action of secreted reductases in the surrounding medium. Efficient strain screening is therefore essential to achieve high reduction yields and controlled nanoparticle characteristics. Marine-derived bacteria are of particular interest due to their adaptation to high salinity, metal exposure, and oxidative stress. Harbor environments, such as the Eastern Alexandria Port, harbor metal-adapted microbiota capable of tolerating oxyanion stress and potentially exhibiting enhanced selenite reductase activity. Despite the growing number of studies on microbial SeNP biosynthesis, several critical gaps remain. Most reports focus on either physicochemical characterization or biological evaluation, with limited integration among systematic strain screening, nanoparticle characterization, and antimicrobial assessment. In addition, although several studies have described SeNP biosynthesis using *Bacillus* species, limited attention has been given to halotolerant marine strains and their capacity to sustain efficient nanoparticle production under saline conditions. Moreover, the relationship among environmental origin, halotolerance, and the efficiency of extracellular nanoparticle synthesis remains underexplored. These limitations hinder a comprehensive understanding of how strain-specific properties influence nanoparticle functionality and application potential (Kadeřábková et al. 2024; Yasir and Zaki 2024; Asaad et al. 2025). To address these gaps, the present study explores a halotolerant marine bacterial strain as a sustainable platform for the biosynthesis of extracellular SeNPs under controlled salinity conditions. The novelty of this work lies in combining strain screening, controlled biosynthesis, physicochemical characterization, and antimicrobial evaluation within a unified experimental framework, enabling a more comprehensive correlation between microbial physiology and nanoparticle properties. The objectives of this study were to (i) isolate and screen marine bacterial strains for their ability to reduce selenium oxyanions, (ii) evaluate extracellular SeNP biosynthesis under optimized salinity conditions, (iii) characterize the produced nanoparticles using multiple analytical techniques, and (iv) assess their antimicrobial activity against selected pathogenic strains. This approach offers several advantages, including eco-friendly synthesis, the use of halotolerant bacteria adapted to extreme environments, and the production of biologically capped nanoparticles with enhanced stability and bioactivity. In the present study, a new halotolerant *Bacillus sonorensis* 2MNHR strain was isolated from Mediterranean seawater samples collected at Eastern Alexandria Port. The produced nanoparticles were characterized using nine complementary analytical techniques, including 16S rRNA-based identification of the producing strain, and their antimicrobial activity was assessed through inhibition assays and MIC determination against six pathogenic strains. This integrated workflow links environmental isolation, nanoparticle characterization, and antimicrobial evaluation to support the sustainable production of SeNPs for potential applications in combating antimicrobial resistance.

## Materials and methods

### Chemicals and reagents

All chemicals used in this study were of analytical grade. Sodium selenite (Na_2_SeO_3_, ≥ 99% purity), sodium selenate (Na_2_SeO_4_, ≥ 98% purity), sodium chloride (NaCl, ≥ 99.5% purity), chloroform (≥ 99% purity), and ethanol (70% *v*/*v*) were obtained from standard commercial suppliers and used without further purification. Deionized water was used throughout all experiments.

### Sample collection and site description

Seawater samples were collected from the Mediterranean Sea at Alexandria Harbor, Alexandria, Egypt (latitude 31° 11′ 36.9492′ N and longitude 29° 52′ 34.5252′ E). The sampling was performed in summer at an ambient temperature of approximately 37 °C. All sampling containers, instruments, and materials were thoroughly sterilized prior to collection to prevent external contamination. Samples were transported aseptically to the laboratory for immediate processing.

### Isolation, purification, and storage of bacterial strains

The seawater sample was serially diluted, plated on Luria–Bertani (LB) agar supplemented with 3.5% (*w*/*v*) NaCl, and incubated aerobically at 37 °C. To ensure culture purity, each isolate was further purified by repeated streaking on LB agar. The purified isolates were maintained on LB agar slants and stored at 4 °C for short-term use. The bacterial strain *B. sonorensis* 2MNHR used in this study is currently maintained in the authors’ laboratory culture collection and is available from the corresponding author upon reasonable request for research purposes.

### Screening strategy for selenite reduction in liquid and solid media

The ability of each purified bacterial isolate to reduce selenium oxyanions under both liquid and solid culture conditions was evaluated using a stepwise screening approach. For the selenium reduction assays, LB medium was supplemented with 3.5% (*w*/*v*) NaCl to simulate marine salinity conditions. The isolates were inoculated into LB broth supplemented with either 1 mM sodium selenite (Na_2_SeO_3_) or 1 mM sodium selenate (Na_2_SeO_4_). The broth cultures were incubated aerobically at 37 °C with shaking at 150 rpm (Avendaño et al. 2016). Cultures were visually monitored for color changes for up to 72 h, with the appearance of a red coloration serving as a qualitative indicator of elemental selenium (Se^0^) formation. Similarly, to evaluate selenium reduction under solid growth conditions, isolates were streaked onto LB agar plates supplemented with either 1 mM sodium selenite or 1 mM sodium selenate (Spyridopoulou et al. 2021). The plates were checked for visible color development daily, and isolates exhibiting red pigmentation were considered likely positive for selenite reduction and were selected for further analysis.

A supernatant-based assay was performed on the isolate that showed a positive response in whole-cell cultures to determine whether selenium reduction occurred intracellularly or extracellularly. The selected isolate was grown in LB broth for 24 h under the same conditions. The bacterial cells were then separated from the culture medium by centrifugation. The resulting cell-free supernatant was aseptically supplemented with sodium selenite to a final concentration of 1 mM and incubated for up to 72 h under the same conditions. The involvement of extracellular components in selenium reduction was inferred based on the presence or absence of red coloration in the supernatant (Kora 2018).

### Salt tolerance assay and growth kinetics

Salt tolerance of the selected bacterial isolate was assessed by growing the strain in LB broth supplemented with increasing concentrations of NaCl (1%, 2%, 3.5%, and 5%, *w*/*v*). LB broth containing 1% NaCl served as the control condition. A concentration of 3.5% NaCl was included to approximate the average salinity of seawater (~ 3.5% dissolved salts), as reported by the National Oceanic and Atmospheric Administration (Li et al. 2021). An overnight culture was inoculated into fresh media and adjusted to an initial optical density (OD_600_) of approximately 0.05. Cultures were shaken and incubated at 37 °C. Bacterial growth was monitored by measuring optical density at 600 nm (OD_600_) at 2-h intervals for a total of 30 h. Growth curves were generated by plotting the OD_600_ values versus incubation time (Omotoyinbo and Omotoyinbo 2016). The maximum optical density (OD_max_) and the time required to reach the stationary phase were determined for each salinity condition to assess the effect of salt concentration on bacterial growth performance. A NaCl concentration of 1% was used for all subsequent selenite reduction and SeNP biosynthesis assays.

### Molecular identification of isolates based on 16S rRNA gene sequencing

Genomic DNA was extracted from the selected bacterial isolates according to the protocol described by Joseph Sambrook (Sambrook 1989). The nearly complete 16S rRNA gene was amplified by polymerase chain reaction (PCR) using universal bacterial primers (Table 1). Each 50-µL PCR reaction mixture contained 30 pmol of each primer, 10 ng of chromosomal DNA, 200 µM of each dNTP, 2.5 U of Taq DNA polymerase, and the supplied reaction buffer. Amplification was performed for 30 cycles under the following conditions: denaturation at 94 °C for 1 min, annealing at 55 °C for 1 min, and extension at 72 °C for 2 min. PCR products were analyzed by agarose gel electrophoresis (Ausubel et al. 1992). The amplified fragments were purified using the QIAquick PCR Purification Kit (Qiagen). Sequencing reactions were carried out using the ABI PRISM BigDye Terminator Cycle Sequencing Kit and analyzed on an ABI PRISM 377 DNA Sequencer (PerkinElmer). The same primers used for amplification were employed for sequencing. Sequence similarity searches were performed using the BLAST program available at the National Center for Biotechnology Information (NCBI). Phylogenetic trees were constructed using MEGA 12, including a rectangular tree of representative isolates and a circular tree of selected taxa (Kumar et al. 2024). The tree visualization and annotation were performed within the same software environment, with further refinement in iTOL v6 (Letunic and Bork 2024).

**Table 1:** Primers used for 16S rRNA gene amplification

Primer name	Sequence (5′ → 3′)	Reference
27 F (forward)	AGAGTTTGATCCTGGCTCAG	(Devereux and Willis 1995)
1492R (reverse)	ACGGTTACCTTGTTACGACTT	

### Purification of biogenic SeNPs

The biogenic SeNPs were separated from the reaction mixture by centrifugation at 4000 rpm for 10 min, and the resulting pellet was collected. To remove residual biological impurities and matrix metabolites associated with the nanoparticles, the pellet was washed sequentially with chloroform, 70% ethanol, and sterile distilled water, yielding purified SeNPs (Pouri et al. 2018; Tabibi et al. 2023; Satpathy et al. 2024). Finally, the nanoparticles were dried overnight at 40 °C for subsequent analysis.

### Characterization of biogenic SeNPs

#### Ultraviolet-visible spectroscopy

The purified biogenic SeNP suspension was dispersed in distilled water, and its absorbance spectra were recorded over a wavelength range of 200–800 nm using an ultraviolet-visible (UV-Vis) spectrophotometer (T80, PG Instruments, UK) (Cheah et al. 2025). Furthermore, parallel UV-Vis spectral analysis was performed on the bacterial supernatant, using the corresponding uninoculated culture medium as the blank control, under identical experimental conditions. All measurements were carried out in triplicate (Bharathi et al. 2020; Tabibi et al. 2023).

#### Fourier-transform infrared spectroscopy

The biomolecules and functional groups associated with the bioreduction of SeNPs were characterized using Fourier-transform infrared (FTIR) spectroscopy (Ranjitha and Ravishankar 2018). A vacuum-dehydrated sample was mixed with KBr pellets, and the infrared spectra were recorded over the range of 450–4000 cm^−1^ using an FTIR spectrophotometer (FTIR spectrum version 10.5.3, PerkinElmer, USA). The spectra were plotted as wavenumber (cm⁻^1^) versus transmittance (%) (Chan et al. 2023; Lim et al. 2025). FTIR analysis was performed on bacterial supernatant, precursor sodium selenite, and biogenic SeNPs to confirm the reduction process and to identify the functional groups and biomolecules involved in the reduction, capping, and stabilization of SeNPs.

#### Zeta potential

The zeta potential of biogenic SeNPs was measured by dispersing 2 mg of SeNPs in 2 mL of deionized water and sonicated for 10 min, with the average of three measurements recorded using a Malvern Zetasizer Nano ZS analyzer (Malvern Instruments, Malvern, UK) (Ullah et al. 2021; Refaat et al. 2024).

#### X-ray diffraction

The crystalline structure of the biogenic SeNPs was determined by X-ray diffraction (XRD) using a Phaser XRD-D2 powder X-ray diffractometer, equipped with Cu-Kα radiation, throughout a range of Bragg angles θ (10° ≤ 2θ ≤ 90°), operated at 30 kV and 10 mA (Ramachandran et al. 2023; Liang et al. 2025).

#### Scanning electron microscopy coupled with energy-dispersive X-ray spectroscopy (SEM–EDX)

The morphology and particle size of the biogenic SeNPs were examined using a scanning electron microscope (SEM) (JEOL Ltd., Tokyo, Japan). The sample was prepared by dispersing the nanoparticle powder in deionized water and sonicated for 30 min to ensure a homogeneous suspension. A drop of the suspension was placed on a clean glass slide and allowed to air-dry, forming a thin layer suitable for SEM imaging.

Additionally, compositional analysis was conducted using the same integrated energy-dispersive X-ray spectroscopy (EDX) system, operated at an accelerating voltage of 20 kV, which enabled the qualitative and quantitative determination of elemental constituents and confirmed the presence of elemental selenium in the nanoparticles.

#### Transmission electron microscopy

Transmission electron microscopy (TEM) analysis was used to determine the morphology and size of biogenic SeNPs. An aqueous suspension of SeNPs was ultrasonically dispersed, and a few drops were deposited on carbon-coated copper TEM grids and subsequently dried at room temperature (Shakibaie et al. 2010; Bahrami et al. 2012). Micrographs were obtained using a TEM (model JEM-1400 Plus, JEOL Corp, Tokyo, Japan) operated at an accelerating voltage of 50 kV.

### Gas chromatography–mass spectrometry analysis of bacterial extract

Gas chromatography–mass spectrometry (GC–MS) analysis was performed to identify the bioactive compounds present in the bacterial extract. The analysis was conducted using a GC-TSQ 8000 Evo mass spectrometer (Thermo Scientific, Austin, TX, USA) equipped with a TG-5MS capillary column (30 m × 0.25 mm internal diameter × 0.25 µm film thickness). The oven temperature was initially set at 60 °C and then increased at a rate of 5 °C/min to 250 °C with a holding time of 2 min, followed by a further increase to 300 °C at 30 °C/min. The injector temperature was maintained at 270 °C. Helium was used as the carrier gas at a constant flow rate of 1 mL/min. A sample volume of 1 µL (diluted extract) was injected automatically using an AS3000 autosampler in split mode. The solvent delay was set to 4 min. Mass spectra were obtained using electron ionization (EI) at 70 eV over a mass scan range of *m*/*z* 50–650 in full scan mode. The ion source temperature was maintained at 200 °C, while the transfer line temperature was set at 280 °C.

### Antimicrobial activity

The antimicrobial activity of the biogenic SeNPs was assessed using the agar well-diffusion method at a concentration of 5 mg/mL against the Gram-positive bacteria, *B. subtilis* (ATCC 6633) and *Staphylococcus aureus* (ATCC 25923), and the Gram-negative bacteria, such as *P. aeruginosa* (ATCC 15692), *Aeromonas hydrophila* (ATCC 13037), and *E. coli* (ATCC 8739). Additionally, the antifungal activity of the suspension was assessed against a clinical isolate of *Candida albicans*. These specific strains were selected based on their clinical significance. Fresh bacterial cultures were grown overnight in LB broth at 37 °C and adjusted to a 0.5 McFarland standard (~ 10^8^ CFU/mL). The standardized suspension was then aseptically spread onto LB agar plates. Wells of 7 mm diameter were aseptically punched into the agar using a sterile cork borer, and 100 µL of the SeNPs suspension (5 mg/mL) was introduced into each well. Gentamicin was employed as a positive control. The plates were incubated at 37 °C for 24 h. The inhibition zone diameter was used as a quantitative indicator of antimicrobial efficacy. All experiments were performed in triplicate, and the results were expressed as mean ± standard deviation (SD).

#### Minimum inhibitory concentration determination

The MIC of the SeNPs was determined using the broth microdilution method in 96-well microtiter plates in accordance with the Clinical and Laboratory Standards Institute guidelines (CLSI) (Clinical and Laboratory Standards Institute 2014). Each well contained 100 μL of LB broth followed by 100 μL of the SeNP suspension. Twofold serial dilutions were prepared to obtain final concentrations ranging from 2500 to 5 μg/mL (Ghallab et al. 2025). Bacterial suspensions were adjusted spectrophotometrically to an OD_600_ equivalent to the 0.5 McFarland standard and further diluted to obtain the recommended inoculum density. Wells containing untreated bacterial cultures served as positive growth controls, while media-only wells were used as negative controls. Plates were incubated at 37 °C for 24 h, and the MIC was defined as the lowest concentration that completely inhibited visible bacterial growth (Naga et al. 2025a; Mohammed et al. 2026).

#### Minimum bactericidal and fungicidal concentration determination

Following MIC determination, 10-µL aliquots from wells showing no visible growth were subcultured onto fresh LB agar plates and incubated at 37 °C for 24 h. The minimum bactericidal concentration (MBC) and minimum fungicidal concentration (MFC) were defined as the lowest concentrations resulting in no visible colony formation, indicating complete microbial killing. The MBC/MIC and MFC/MIC ratios were used to determine whether the antimicrobial activity was bactericidal/fungicidal or bacteriostatic/fungistatic. SeNPs were considered bactericidal/fungicidal when the MBC/MIC ratio was ≤ 4 and bacteriostatic/fungistatic when the ratio was > 4 (Ishak et al. 2025).

### Statistical analysis

All experiments were performed in triplicate unless otherwise stated, and characterization analyses were conducted on independently prepared nanoparticle batches to ensure reproducibility. Results are expressed as mean ± standard deviation (SD). Statistical analysis of inhibition zone diameters was performed using two-way analysis of variance (ANOVA) followed by Tukey’s multiple comparisons test using GraphPad Prism 8 software. Differences were considered statistically significant at ^*^p < 0.05, ^**^p < 0.01, and ^***^p < 0.001.

## Results

### Isolation of bacterial strains

Ten morphologically distinct bacterial isolates were successfully isolated from Alexandria Harbor, Mediterranean Sea, Egypt, following cultivation on LB agar plates supplemented with 3.5% NaCl. All isolates exhibited aerobic growth under these conditions and were subsequently screened for their ability to reduce selenium oxyanions.

### Screening of bacterial isolates for selenite and selenate reduction

**Fig. 1:**
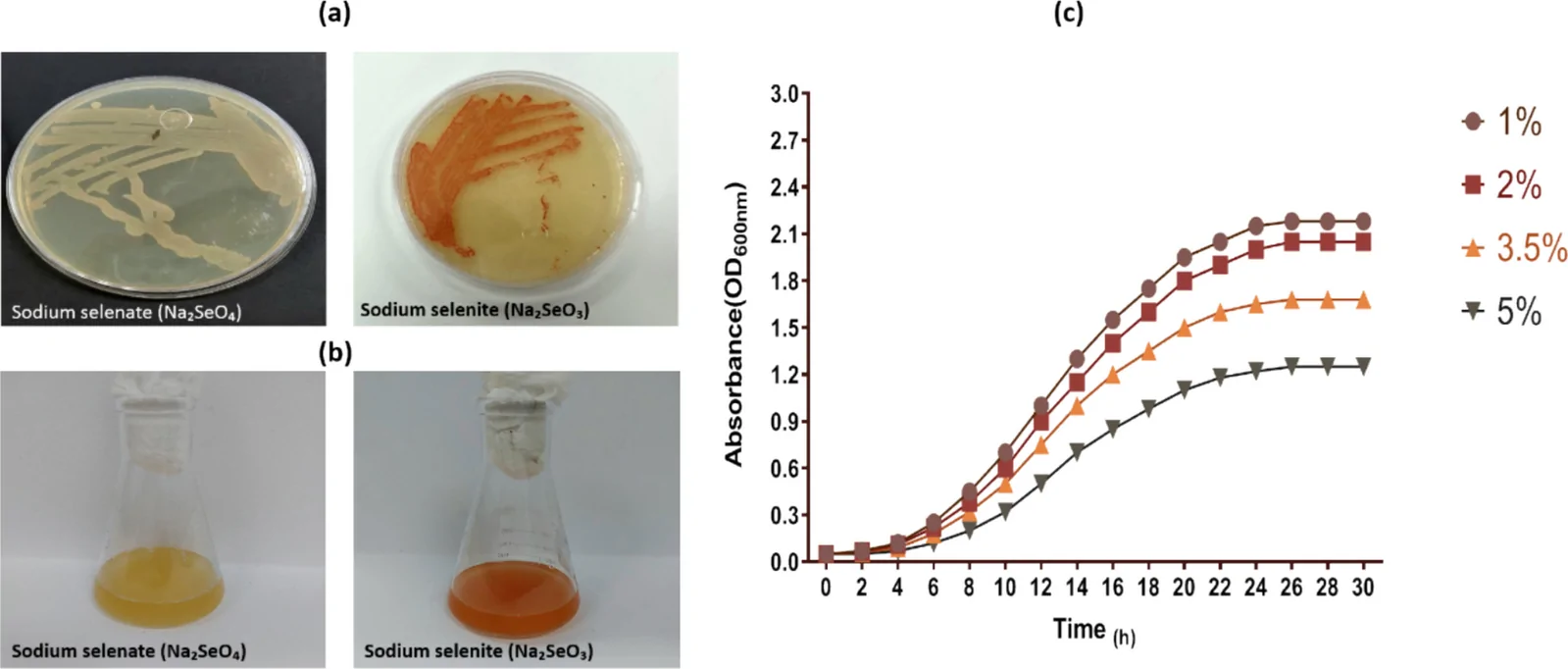
**a** Solid culture and **b** liquid culture showing red coloration indicative of sodium selenite reduction, while no visible reduction was observed in sodium selenate-containing media. **c** Growth kinetics of the selected isolate at different NaCl concentrations (1–5%), demonstrating optimal growth at 1% NaCl

All ten isolates were screened for their ability to reduce sodium selenite (Na_2_SeO_3_) and sodium selenate (Na_2_SeO_4_) under both liquid and solid culture conditions. Among the tested isolates, only one isolate developed a distinct red coloration within 72 h in LB broth containing 1 mM sodium selenite, indicating the formation of elemental selenium. The same isolate exhibited red pigmentation on selenite-supplemented LB agar plates (Fig. 1a, b), whereas the remaining isolates showed no visible color change in either medium. In contrast, none of the isolates demonstrated detectable color development in the presence of sodium selenate throughout the incubation period.

### Supernatant-based assay following whole-cell screening

The isolate that consistently showed sodium selenite reduction in both liquid and solid media was subjected to a supernatant-based assay after whole-cell screening. Following 24 h of aerobic cultivation in LB broth, the bacterial cells were pelleted by centrifugation to obtain cell-free supernatants. The supernatants were then aseptically supplemented with sodium selenite to a final concentration of 1 mM and incubated for up to 72 h under identical conditions. Following incubation, a noticeable red coloration appeared in the cell-free supernatant, indicating that selenite reduction occurred independently of intact bacterial cells. These findings suggest that selenium reduction occurs via an extracellular mechanism. Based on its consistent performance in both the whole-cell and supernatant-based assays, this isolate was selected for subsequent SeNP biosynthesis and further physiological and molecular characterization.

### Characterization of the selected selenite-reducing isolate

#### Salt tolerance assay of the selected isolate

The selected isolate exhibited optimal growth in medium supplemented with 1% NaCl. Growth progressively decreased with increasing salinity; however, measurable growth was still observed at 5% NaCl. These findings indicate that the isolate is halotolerant rather than halophilic (Fig. 1c).

#### Molecular identification based on 16S rRNA gene sequencing

**Fig. 2:**
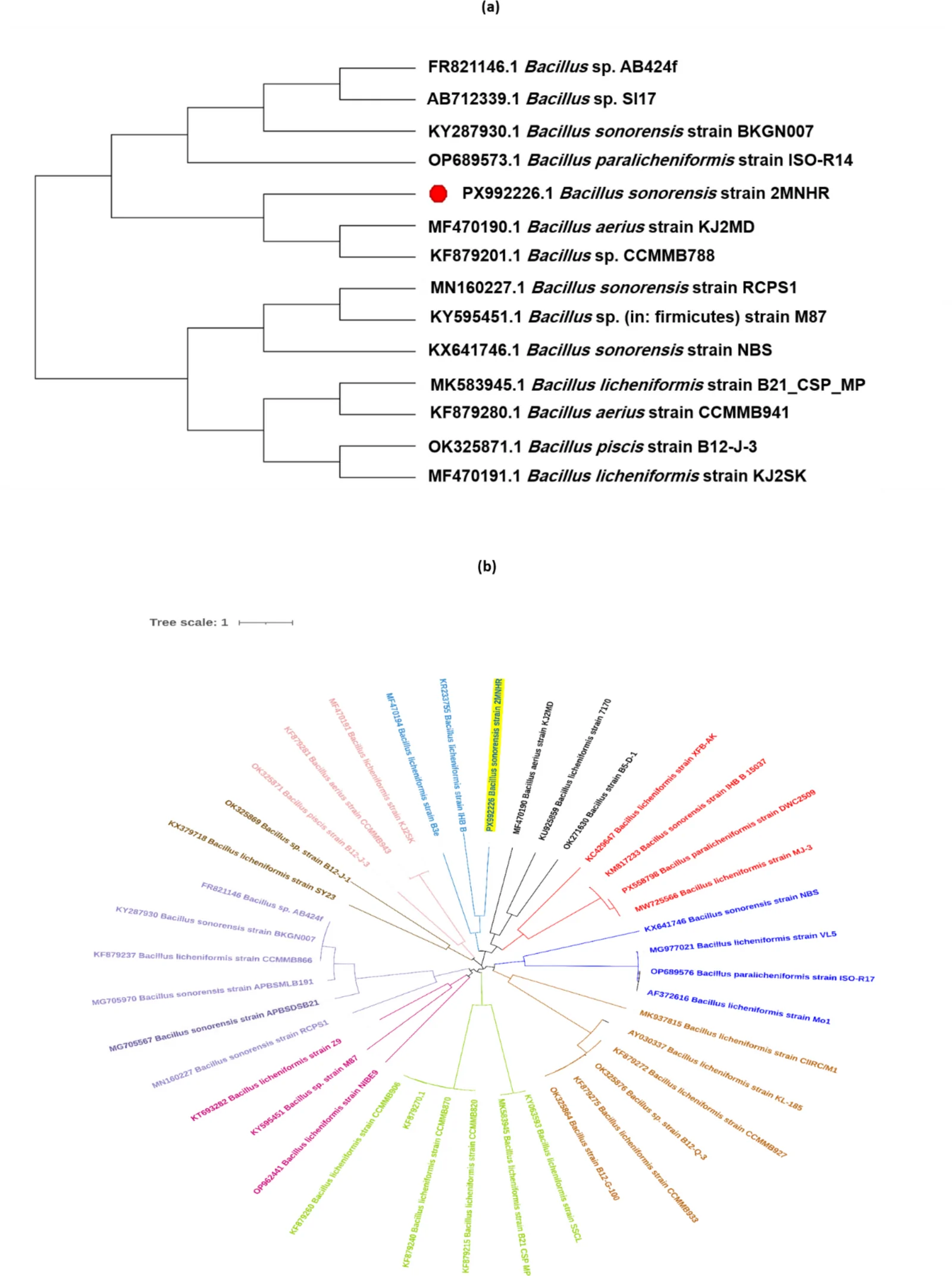
Phylogenetic trees constructed based on partial 16S rRNA gene sequences. **a** Rectangular tree including 14 representative taxa. **b** Circular tree comprising 40 taxa. The selected isolate (*B. sonorensis* 2MNHR) is indicated by a filled circle. Trees were generated using the neighbor-joining method with 1000 bootstrap replicates in MEGA 12 and refined in iTOL v6

PCR amplification of the 16S rRNA gene from the selected isolate yielded a single clear band as verified by agarose gel electrophoresis. The purified amplicon was successfully sequenced, and the resulting partial 16S rRNA gene sequence was deposited in the GenBank database under accession number PX992226. Sequence similarity analysis using the BLAST algorithm available at the NCBI revealed 100% identity with *B. sonorensis* sequences. Based on the highest sequence identity and phylogenetic clustering, the isolate was identified as *B. sonorensis* and designated as *B. sonorensis* 2MNHR. Phylogenetic analysis was performed using MEGA 12. The constructed phylogenetic trees demonstrated that the isolate clustered robustly with reference strains of *B. sonorensis* and was clearly separated from closely related species. The topology was consistent across both rectangular (14 taxa) and circular (40 taxa) tree layouts, supporting the taxonomic placement of the isolate within the genus *Bacillus* (Fig. 2).

### Characterization of biogenic SeNPs

#### Ultraviolet-visible analysis

**Fig. 3:**
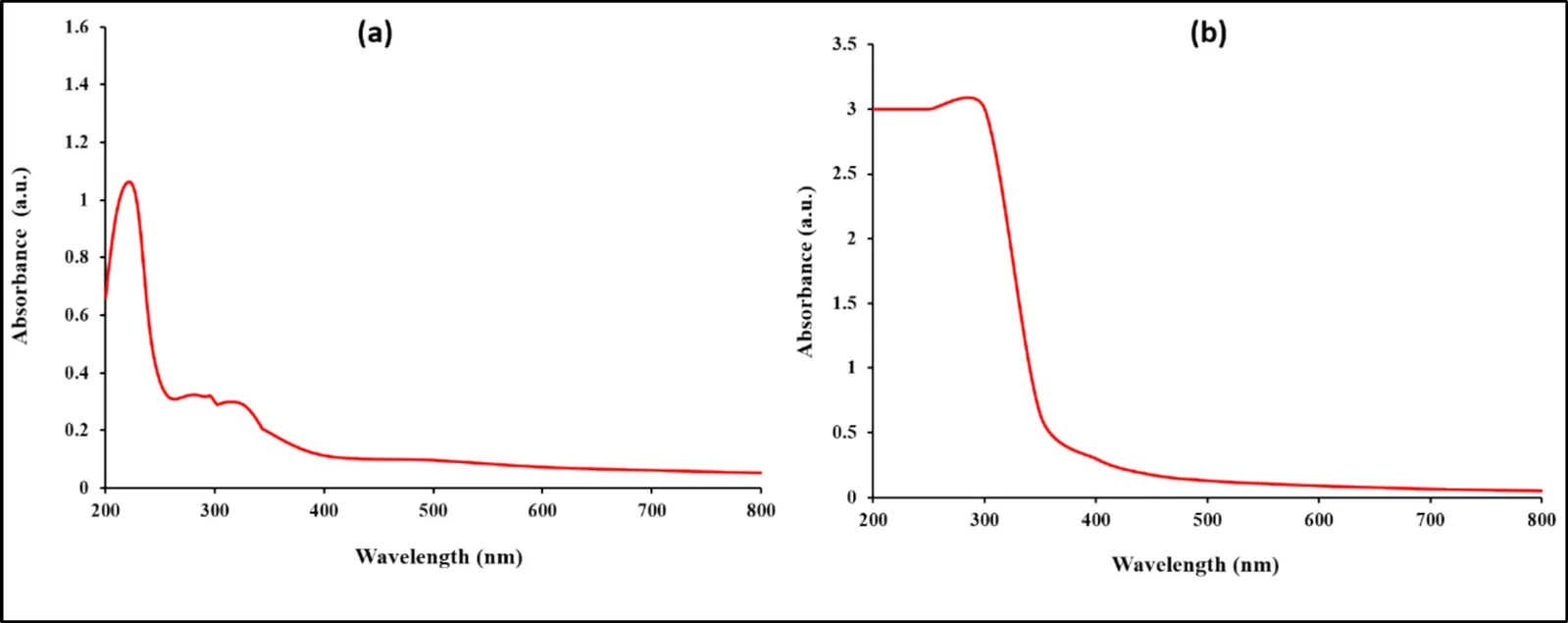
UV-Vis spectrum of **a** biogenic SeNPs and **b** bacterial filtrate by *B. sonorensis* 2MNHR after 72 h at 37 °C

The UV-visible spectral analysis of the bacterial supernatant and the synthesized SeNPs (Fig. 3) provided robust evidence for the successful biogenic formation of nanoparticles. Initially, the biosynthesis of SeNPs by *B. sonorensis* 2MNHR was visually indicated by a distinct color change of the culture medium from pale yellow (control) to orange–red, suggesting the reduction of sodium selenite to elemental selenium (Se^0^). The UV-Vis spectrum of the bacterial filtrate exhibited a broad absorption band in the 200–300 nm range, characterized by high absorbance values (~ 3) (Fig. 3b), reflecting the presence of diverse biomolecules in the bacterial supernatant. In contrast, the UV-Vis spectrum of the purified SeNPs showed a distinct absorption peak at 226 nm with a lower absorbance value (~ 1) (Fig. 3a). Furthermore, the UV-Vis spectrum of the purified SeNPs displayed a prominent absorption peak within the 200–400 nm region, accompanied by a minor shoulder around 326 nm. These spectral features are characteristic of SeNPs and support the successful biotransformation of sodium selenite into nanoscale elemental selenium (Se^0^).

#### Fourier-transform infrared spectroscopy analysis

**Fig. 4:**
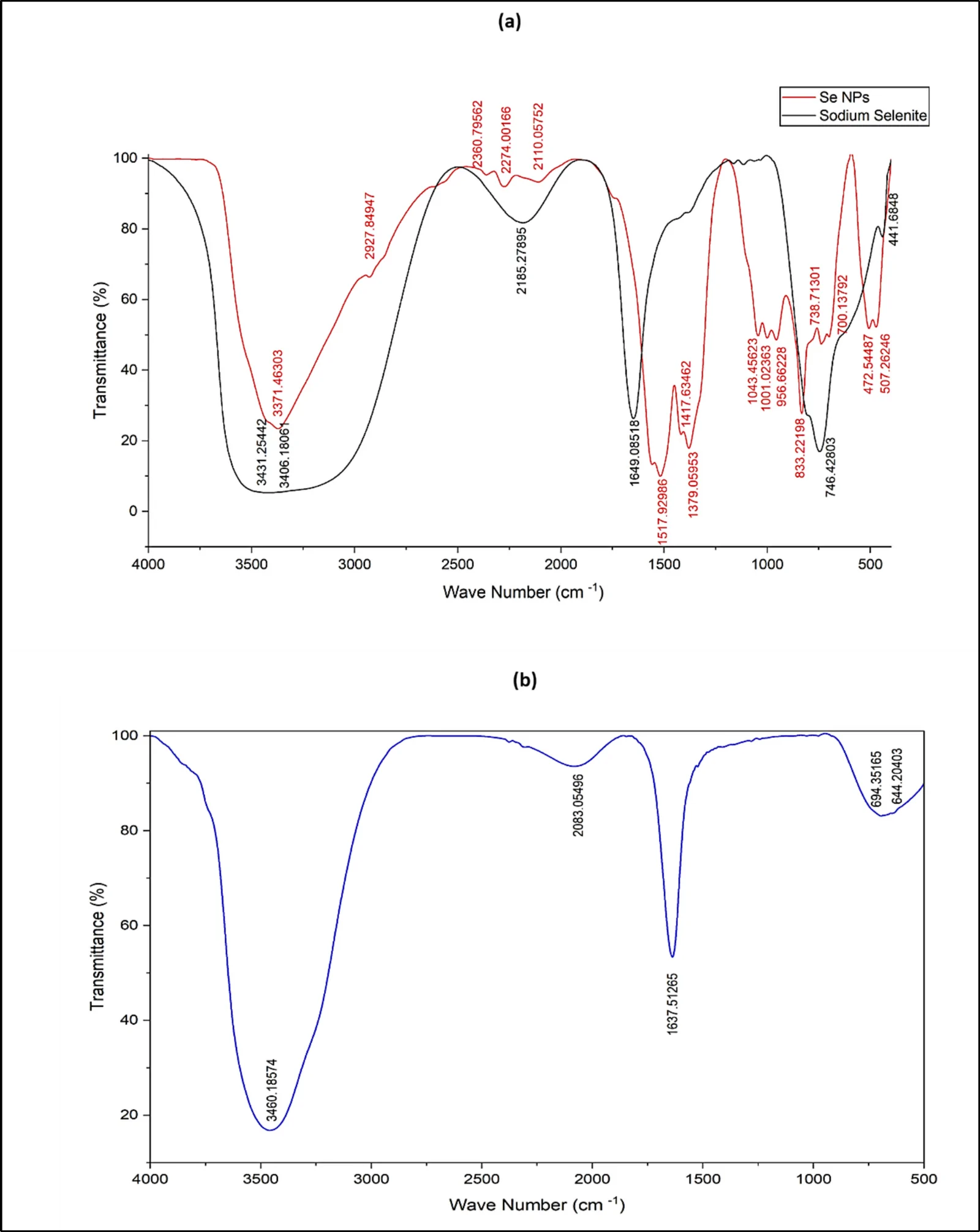
Fourier transform infrared spectroscopy of **a** biogenic SeNPs and **b** bacterial filtrate by *B. sonorensis* 2MNHR

The FTIR spectrum of the bacterial supernatant (Fig. 4b) exhibited a broad and intense absorption band at 3460.19 cm⁻^1^, which is typically assigned to O–H and N–H stretching vibrations arising from proteins, peptides, and other extracellular biomolecules. A weak band observed at 2083.05 cm⁻^1^ is likely associated with weakly bound functional groups in the supernatant, possibly reflecting C–H stretching vibrations of aliphatic chains characteristic of lipid or protein components. In addition, a distinct peak at 1637.51 cm⁻^1^ was detected, corresponding to the amide I band of proteins (Saraeva et al. 2022). In the fingerprint region, peaks at 694.35 and 644.40 cm⁻^1^ were observed, which can be attributed to the skeletal vibrations of biomolecular components and low-frequency modes of organic constituents. For comparison, the FTIR spectrum of the precursor sodium selenite (Fig. 4a) exhibited its characteristic absorption bands at 441, 746, 1649, 2185, 3406, and 3431 cm⁻^1^.

In contrast, the biosynthesized SeNPs showed a marked reduction in the intensity of several precursor-related bands, accompanied by the appearance of new absorption peaks that were absent in the sodium selenite spectrum. A broad band centered at 3371 cm⁻^1^ was observed, attributable to O–H and N–H stretching vibrations and indicative of hydroxyl and amine groups commonly associated with proteins (Kamnev et al. 2021). Additional peaks at 1517, 1417, 1379, 1043, 1001, 956, 833, and 507 cm⁻^1^ were also detected, corresponding to amide I and amide II vibrations, C–N stretching, and other protein-related or biomolecular signatures. These spectral shifts and newly emerged bands indicate the involvement of bacterial biomolecules, particularly proteins, in the reduction of sodium selenite and the stabilization of the resulting SeNPs. These findings confirm the successful bioreduction of selenite to elemental selenium (Se^0^) and indicate that bacterial proteins likely play a dual role as both reducing and stabilizing agents during SeNP biosynthesis.

#### Zeta potential analysis

**Fig. 5:**
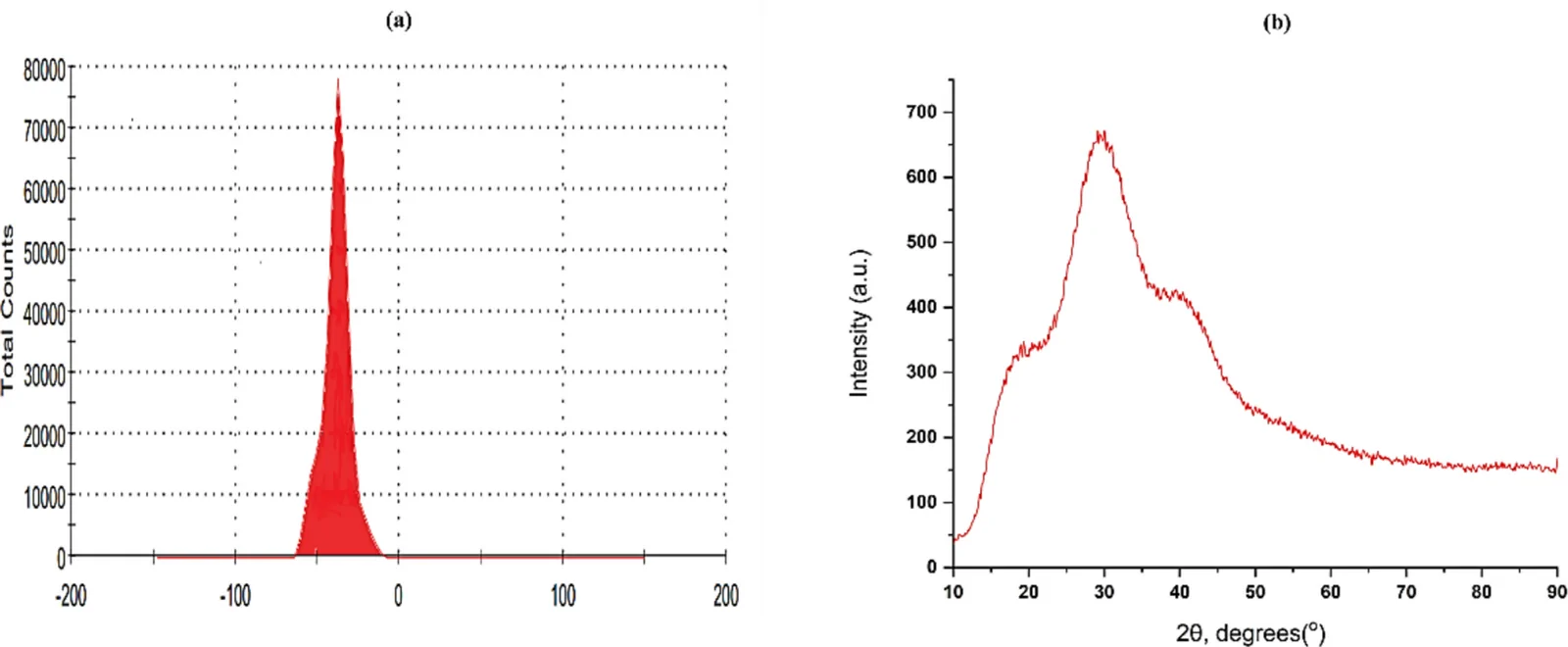
Physicochemical characterization of biogenic SeNPs synthesized by *B. sonorensis* 2MNHR. **a** Zeta potential showing a highly negative surface charge (−38 mV) and strong colloidal stability. **b** XRD pattern with a broad peak at 2θ = 25–35°, indicating an amorphous or poorly crystalline structure

Zeta potential, which reflects the net electrostatic charge on particles in suspension, is a key determinant of colloidal stability. The biogenic SeNPs exhibited a zeta potential of −38 mV (Fig. 5a), indicating high stability within the nanoparticle suspension. In general, colloidal dispersions with absolute zeta potential values greater than ± 30 mV are considered stable, whereas lower values may lead to aggregation and flocculation (Guisbiers et al. 2016). A higher magnitude zeta potential confers stronger electrostatic repulsion between nanoparticles, thereby preventing agglomeration and ensuring long-term colloidal stability (Gunti et al. 2019; Stabnikova et al. 2023; Sadeghi et al. 2025).

#### X-ray diffraction analysis

XRD analysis of the biogenic SeNPs (Fig. 5b) revealed a broad diffraction peak in the 2θ angles (25–35°), confirming an amorphous or poorly crystalline structure. This observation suggests that the biosynthesized SeNPs lack long-range crystalline order, which is typical for biologically produced SeNPs (Kora 2018; Shi et al. 2021; Spyridopoulou et al. 2021; Cinar-Acar 2025).

#### Morphology and elemental analysis of biogenic SeNPs using SEM, TEM, and EDX

**Fig. 6:**
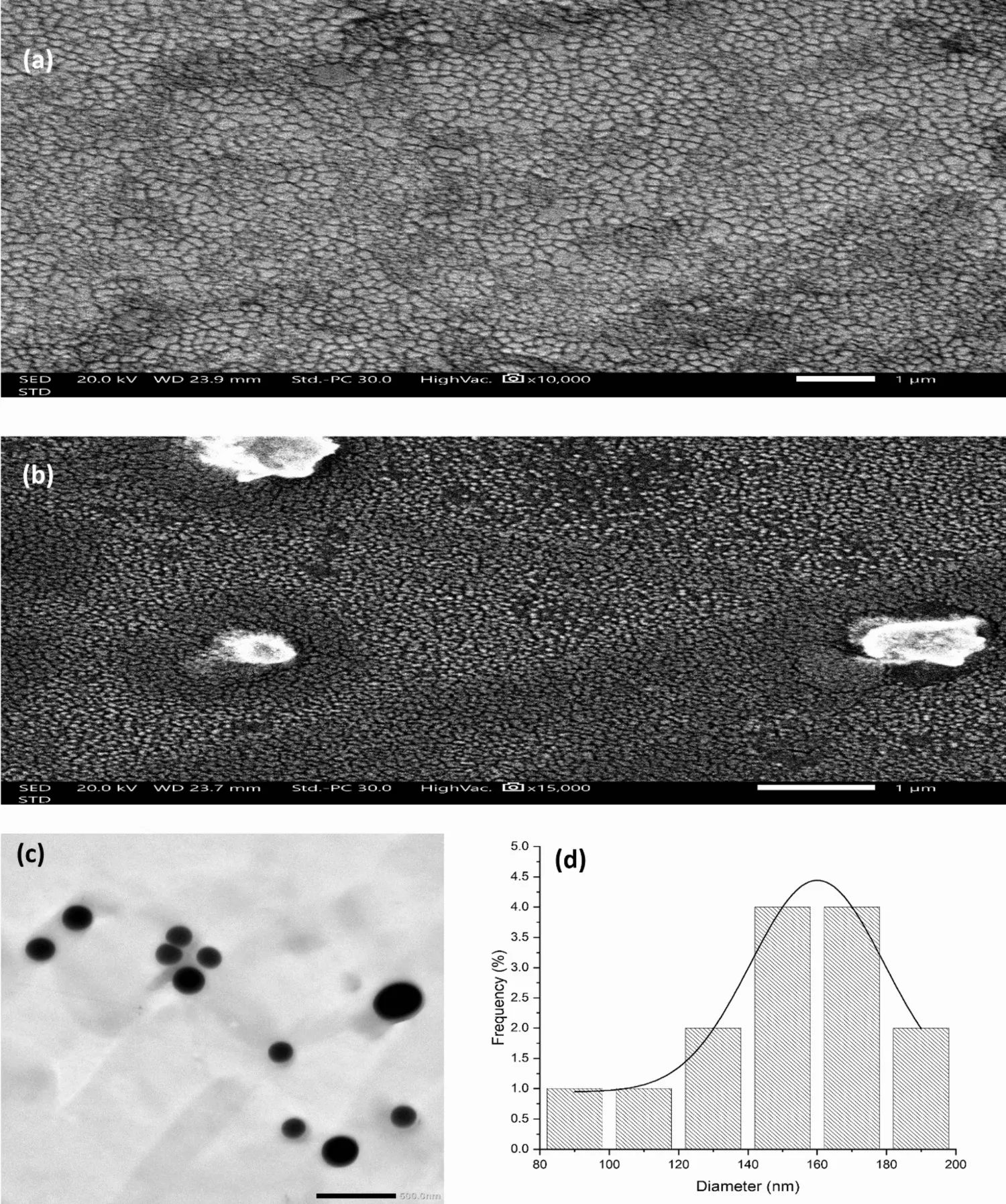
Morphology and composition of biogenic SeNPs from *B. sonorensis* 2MNHR. **a**, **b** SEM images at 10,000 × and 15,000 × showing granular to spherical nanoparticles with occasional aggregation (110–200 nm). **c** TEM image confirming spherical, amorphous structures. **d** TEM-based size distribution with a mean diameter of 160.03 nm

**Fig. 7:**
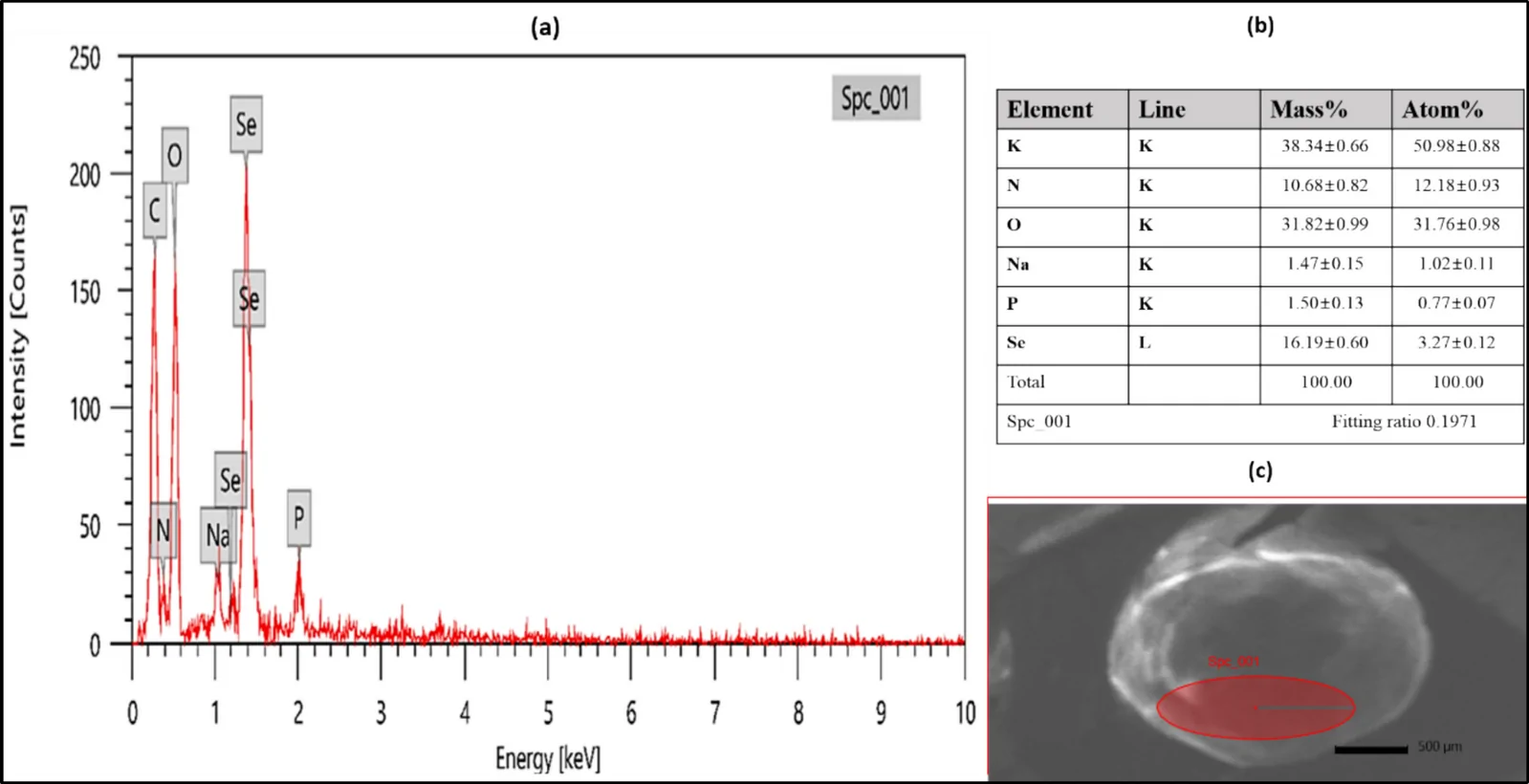
EDX analysis and morphology of biogenic SeNPs by *B. sonorensis* 2MNHR. **a** EDX spectrum showing Se with the SeLα peak at 1.37 keV; C, O, N, P, and Na signals indicate biomolecular capping. **b** The table lists Mass% and Atom% of each element. **c** SEM image of SeNPs (signal: SED; landing voltage: 20.0 kV; WD: 10.0 mm; magnification: × 35; high vacuum)

SEM analysis of the biogenic SeNPs revealed granular-to-spherical nanoparticles, with some degree of aggregation. Images captured at magnifications of 10,000 × and 15,000 × (Fig. 6a, b) indicated particle sizes ranging from 110 to 200 nm. EDX spectroscopy confirmed the elemental composition of the nanoparticles, showing a characteristic SeLα peak at 1.37 keV with an atomic percentage of 16.19%. Additional signals corresponding to C, O, N, P, and Na were detected (Fig. 7), which are likely derived from extracellular macromolecular components such as proteins, polysaccharides, phospholipids, and nucleic acids present in the *B. sonorensis* 2MNHR supernatant. This confirms the role of biological macromolecules in capping and stabilizing the SeNPs (Al-Kordy et al. 2021; Hezema et al. 2025). TEM provided further confirmation of the SeNP morphology (Fig. 6c). The nanoparticles were predominantly spherical and amorphous in structure. Analysis of the TEM images using ImageJ software (https://imagej.net/ij/index.html) allowed for the determination of the particle size distribution, which ranged from 80 to 200 nm, with a mean size of 160.03 nm (Fig. 6d). The TEM observations closely corroborated the SEM findings, confirming the nanoscale dimensions and spherical morphology of the biogenic SeNPs.

### Gas chromatography–mass spectrometry analysis of bacterial extract

**Fig. 8:**
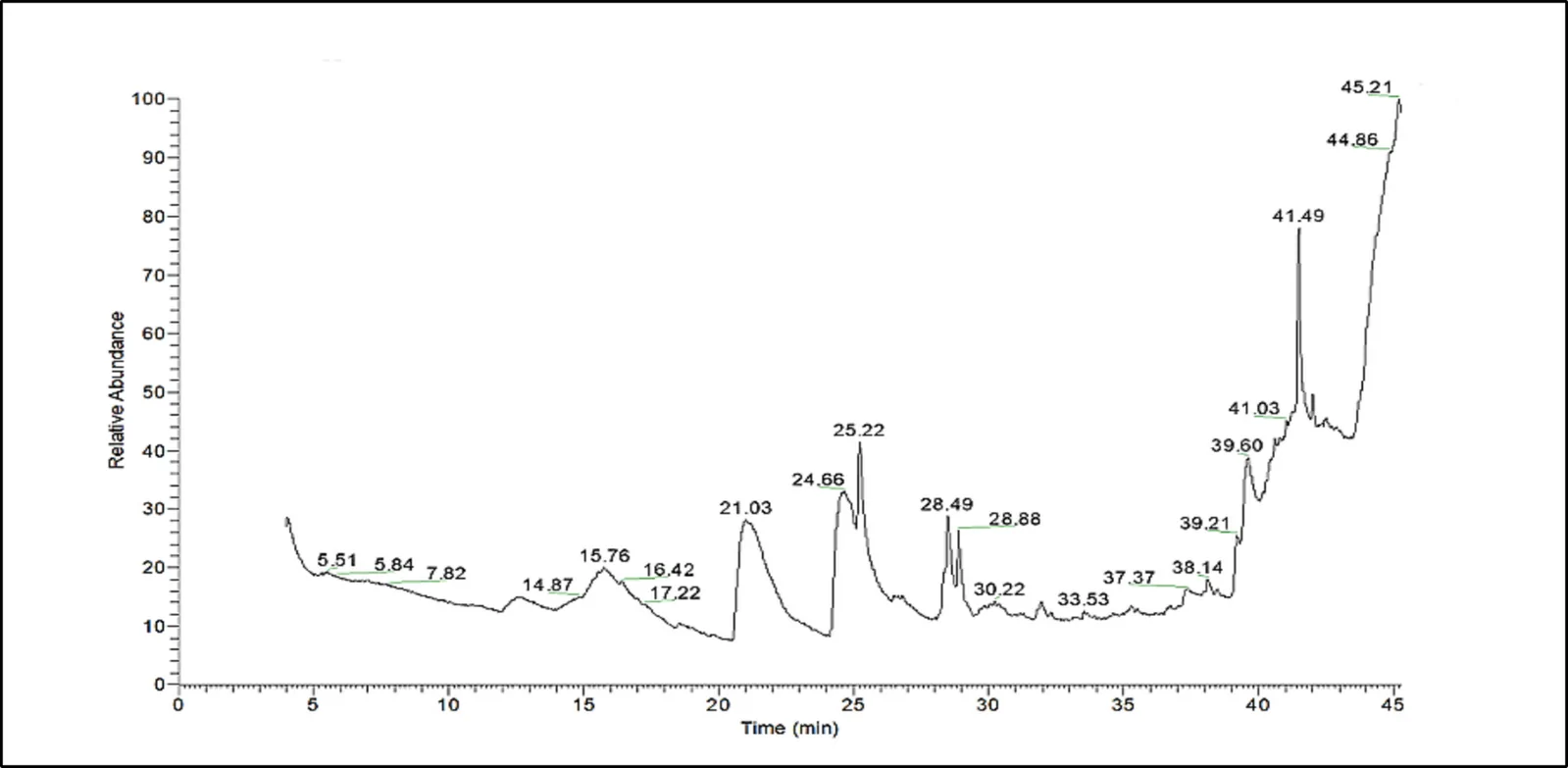
GC–MS chromatogram of the bacterial extract involved in SeNP biosynthesis, showing relative abundance (%) versus retention time (min)

GC–MS analysis of the bacterial extract revealed a rich profile of bioactive metabolites, primarily comprising sterols, fatty acids, aromatic derivatives, and carotene-related molecules. The most abundant compounds were identified at retention times (RT) of 20.96, 24.66, and 25.22 min, representing area percentages (Area %) of 15.74%, 11.34%, and 10.50%, respectively. These major peaks corresponded to ethyl iso-allocholate, 9,12-octadecadienoic acid (Z,Z)-, and acetonitrile, (m-phenoxyphenyl)-, highlighting the dominance of specific organic metabolites essential for biological activity. Notably, the presence of ethyl iso-allocholate and various fatty acid derivatives suggests their dual role as bioreductants and capping agents during SeNP synthesis. Furthermore, the detection of benzaldehyde derivatives and polyunsaturated fatty acids underscores the potent antimicrobial properties of the extract, which likely contribute to the disruption of microbial cell membranes (Fig. 8, Table 2).

**Table 2:** Major compounds identified by GC–MS analysis of bacterial extract

RT (min)	Compound name	Area (%)	Proposed role
20.96	Ethyl iso-allocholate	15.74	Bioreductant and stabilizer
24.66	9,12-Octadecadienoic acid (Z,Z)-	11.34	Antimicrobial agent
25.22	Acetonitrile, (m-phenoxyphenyl)-	10.50	Bioactive intermediate
24.58	9,12-Octadecadienoic acid (Z,Z)-(isomer)	9.68	Capping and stabilizing agent
41.49	1-Heptatriacotanol	8.19	Structural stabilizer
39.59	Carotene, 1,1′,2,2′-tetrahydro	7.48	Antioxidant agent
28.49	Benzaldehyde, 3-phenoxy-	5.76	Antimicrobial effector
28.88	Squalene	4.32	Antioxidant and bioenhancer
41.64	9,12-Octadecadienoic acid derivative	2.35	Synergistic antimicrobial
41.25	Fatty acid ester derivative	1.86	Surface modifier
15.75	Hexadecanoic acid, ethyl ester	1.64	Antibacterial and antifungal
28.34	Phenoxy derivative	1.09	Secondary bioactive
38.13	Vitamin E derivative	0.94	Bioreduction facilitator
16.09	Phytol derivative	0.83	Bioactive terpene

### Antimicrobial activity and minimum inhibitory concentration determination of biogenic SeNPs

**Fig. 9:**
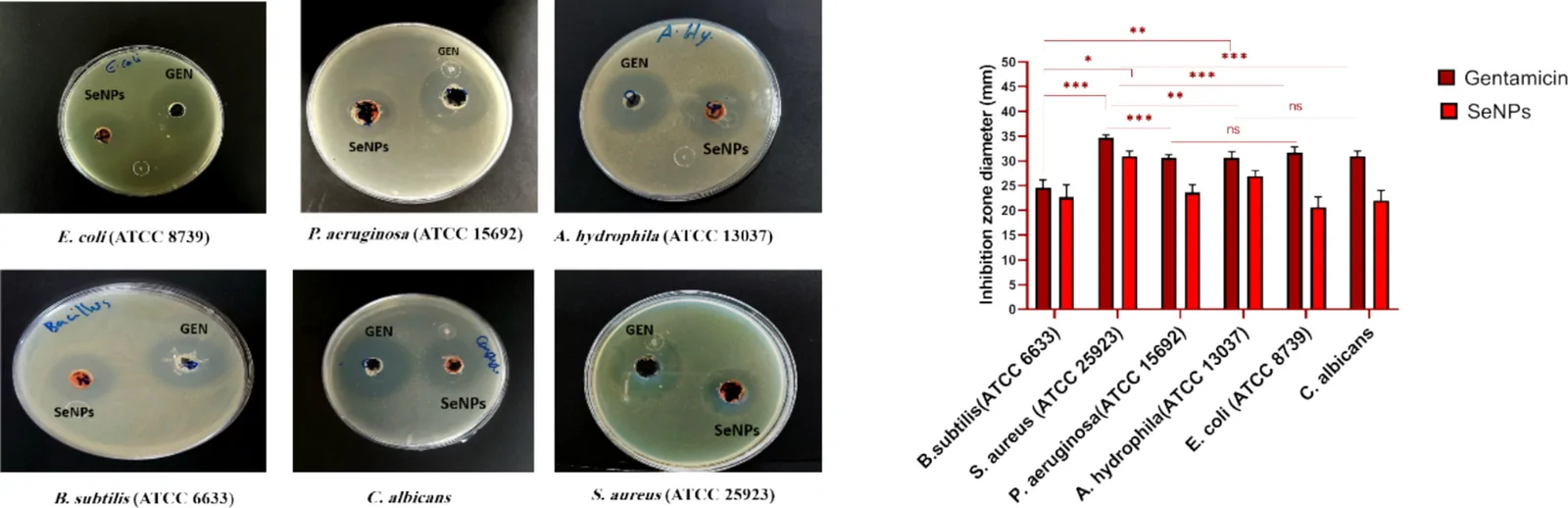
Antimicrobial activity of biogenic SeNPs synthesized by *B. sonorensis* 2MNHR against selected microbial strains. Inhibition zone diameters (mm) are expressed as mean ± SD (*n* = 3). Statistical analysis was performed using two-way ANOVA followed by multiple comparisons test (Tukey’s HSD). Significant differences between groups are indicated as ^*^p < 0.05, ^**^p < 0.01, ^***^p < 0.001, and ns (not significant)

Six microbial strains—five bacterial species (*B. subtilis* ATCC 6633, *S. aureus* ATCC 25923, *P. aeruginosa* ATCC 15692, *A. hydrophila* ATCC 13037, and *E. coli* ATCC 8739) and one fungal strain (*C. albicans*, clinical isolate)—were used to assess the antimicrobial activity of the biogenic SeNPs. The SeNPs exhibited promising antimicrobial activity against all tested strains, with inhibition zone diameters ranging from 23 to 32 mm (Table 3, Fig. 9). Among the tested microorganisms, *S. aureus* exhibited the highest susceptibility, as indicated by the largest inhibition zone diameter (32 mm). This enhanced susceptibility may be attributed to differences in cell wall structure between Gram-positive and Gram-negative bacteria. MIC values were determined for each isolate and ranged from 19.5 to 156.25 µg/mL, indicating varying susceptibility among the tested microorganisms (Table 3). Gentamicin was included as a positive control in all assays and produced the expected inhibition zones, confirming the validity of the assay. These results demonstrate the potential of the biogenic SeNPs as effective antimicrobial agents against both bacterial and fungal pathogens.

**Table 3:** Antimicrobial activity, MIC, MBC (bacteria), MFC (fungi), and MBC/MIC or MFC/MIC ratios of biogenic SeNPs

Microorganisms	Inhibition zone (mm) ± SD	Gentamicin inhibition zone (mm) ± SD	MIC (µg/mL)	MBC (µg/mL)	MBC/MIC	Effect
*B. subtilis* (ATCC 6633)	23 ± 2.51^c^	26 ± 1.53	39	78	2	Bactericidal
*S. aureus* (ATCC 25923)	32 ± 1.00^a^	34 ± 0.58	156.25	312.5	2	Bactericidal
*P. aeruginosa* (ATCC 15692)	25 ± 1.53^b^	31 ± 0.58	156.25	625	4	Borderline bactericidal
*A. hydrophila* (ATCC 13037)	26 ± 1.00^b^	30 ± 1.15	19.5	39	2	Bactericidal
*E. coli* (ATCC 8739)	23 ± 2.08^c^	31 ± 1.15	78.125	156.25	2	Bactericidal
*C. albicans*	24 ± 2.00^c^	30 ± 1.00	19.5	78	4	Borderline fungicidal

Values are expressed as mean ± SD (*n* = 3). Different superscript letters indicate statistically significant differences (*p* < 0.05) according to two-way ANOVA followed by Tukey’s HSD post hoc test

#### Minimum bactericidal and fungicidal concentration and MBC/MIC and MFC/MIC ratios

The bactericidal and fungicidal activities of the biosynthesized SeNPs were further evaluated by determining the MBC for bacterial strains and the MFC for *C. albicans*. The obtained MBC values for the bacterial isolates ranged from 39 to 625 µg/mL, whereas the MFC value for the fungal strain was 78 µg/mL. The calculated MBC/MIC and MFC/MIC ratios were used to assess the nature of antimicrobial activity. Most bacterial strains, including *B. subtilis* ATCC 6633, *S. aureus* ATCC 25923, *A. hydrophila* ATCC 13037, and *E. coli* ATCC 8739, exhibited MBC/MIC ratios of 2, indicating a strong bactericidal effect of the SeNPs. In contrast, *P. aeruginosa* ATCC 15692 showed a ratio of 4, suggesting borderline bactericidal activity. The fungal strain *C. albicans* exhibited an MFC/MIC ratio of 4, indicating a fungistatic tendency rather than a fully fungicidal effect. These findings confirm that the SeNPs predominantly exert bactericidal activity, with relatively lower fungicidal efficiency (Table 3).

#### Statistical analysis of antimicrobial activity

Two-way ANOVA followed by Tukey’s multiple comparisons test revealed significant differences in the antimicrobial activity of the biosynthesized SeNPs among the tested microorganisms. *S. aureus* exhibited significantly higher susceptibility compared to all other tested strains, including *B. subtilis* (*p* < 0.0001), *P. aeruginosa* (*p* = 0.0005), *A. hydrophila* (*p* = 0.0074), *E. coli* (*p* = 0.0001), and *C. albicans* (*p* = 0.0002). Significant differences were also observed between *B. subtilis* and both *P. aeruginosa* (*p* = 0.0178) and *A. hydrophila* (*p* = 0.0011), indicating moderate variation in susceptibility among these strains. However, no significant differences were detected between *B. subtilis* and either *E. coli* (*p* = 0.1072) or *C. albicans* (*p* = 0.0590). Furthermore, comparisons among Gram-negative bacteria showed no statistically significant differences, including between *P. aeruginosa*, *A. hydrophila*, and *E. coli* (*p* > 0.05). Similarly, no significant differences were observed between these bacterial strains and *C. albicans*, suggesting comparable levels of susceptibility. Overall, these findings demonstrate that the antimicrobial efficacy of SeNPs is strongly influenced by microbial species, with *S. aureus* being the most susceptible, while other microorganisms exhibited relatively similar responses under the tested conditions (Fig. 9).

## Discussion

The present study describes the isolation of a halotolerant marine strain, *B. sonorensis* 2MNHR, capable of extracellular biosynthesis of SeNPs. This work integrates physicochemical characterization with antimicrobial performance to provide a comprehensive interpretation of SeNP functionality. Although the strain was isolated from a saline marine environment, it exhibited optimal growth at moderate salinity (1% NaCl), indicating physiological adaptability rather than strict halophilicity. This growth pattern is characteristic of halotolerant *Bacillus* species, which are able to grow across a broad salinity range without requiring high salt concentrations. For example, *B. subtilis* HG-15 has been reported to tolerate up to 30% NaCl (Ji et al. 2022). Such flexibility is typically attributed to efficient osmoadaptation mechanisms, including the accumulation of compatible solutes and regulation of membrane transport systems. Therefore, the ability of our isolate to grow under moderate salinity, while tolerating selenite stress, highlights its suitability for biotechnological applications in environments with fluctuating salt concentrations. Importantly, this is not the first report of *B. sonorensis* lineage in Egypt. Related strains have previously been isolated from Marsa Matrouh (Abdelnasser and Abu-Shahba 2024), which is also located along the Mediterranean coast, as well as from the hot spring at Pharaoh’s bath in Sinai (Saeed et al. 2020). This geographical distribution strongly suggests that *B. sonorensis* strains are well established across Egypt, particularly within Mediterranean and arid coastal ecosystems. The repeated isolation of this species from saline and environmentally challenging habitats further supports the notion that this lineage is highly adapted to extreme or fluctuating conditions. This resilience is consistent with the biology of the genus *Bacillus*, whose members are well-known endospore formers. Endospore formation enables survival under nutrient limitation, osmotic stress, desiccation, and other harsh environmental pressures. Consequently, their persistence in saline marine habitats and their ability to cope with metalloid stress, such as selenite exposure, are ecologically and physiologically advantageous.

Among the screened isolates, only one strain demonstrated efficient aerobic reduction of sodium selenite to elemental selenium (Se^0^). The rapid transformation of the culture medium from colorless to a characteristic orange–red color provided visual confirmation of colloidal selenium formation. This chromatic shift is widely recognized as a hallmark of microbial selenite bioreduction (Sharma et al. 2014). The observed UV-visible spectral changes provide insight into the role of bacterial metabolites in the biosynthesis of the nanoparticles. The bacterial supernatant exhibited a broad absorption band in the 200–300 nm region with relatively high absorbance (~ 3), reflecting an abundance of biomolecules, including proteins, nucleic acids, and other metabolites. These biomolecules are widely recognized for their dual function as both reducing and stabilizing agents in biologically mediated nanoparticle synthesis. The UV-Vis spectrum of the synthesized SeNPs showed a characteristic absorption peak around 226 nm, which may be associated with nanoscale selenium formation together with contributions from biomolecular components present in the system, accompanied by a marked decrease in absorbance (~ 1). This decrease in absorbance suggests the consumption or structural transformation of these biomolecules during the reduction process (Ramamurthy et al. 2013; Zare et al. 2013; Cai et al. 2018; Shar et al. 2020; Yilmaz et al. 2021; Saravanan et al. 2025; Soheyli et al. 2025). The absorption peak at 226 nm may also indicate a blue shift associated with nanoscale formation and size-dependent optical behavior. The optical band gap was about 2.88 eV, which may be attributed to band tailing and the amorphous nature of the nanoparticles (Ikhmayies and Ahmad-Bitar 2013). These results are consistent with the XRD pattern and suggest that the optical properties are influenced by localized electronic states and biomolecule-mediated surface effects. The FTIR analysis provides strong evidence that the bacterial supernatant actively participated in both the reduction of sodium selenite and the stabilization of the synthesized selenium nanoparticles. The detected O–H, N–H, and amide bands indicate the presence of proteinaceous biomolecules acting as reducing and capping agents. These FTIR findings are in strong agreement with the UV-Vis analysis, confirming the successful reduction process. Similar FTIR patterns were reported for *Bacillus*-mediated SeNPs (Wang et al. 2024). The presence of these functional groups confirms that the nanoparticles are biologically capped, which enhances colloidal stability and prevents uncontrolled aggregation. Electron microscopy analyses further clarified nanoparticle morphology and elemental composition. SEM images revealed predominantly spherical-to-granular nanoparticles with some degree of aggregation, while TEM confirmed their amorphous and spherical structure with a size range of approximately 80–200 nm. This size distribution falls within the typical range reported for biologically synthesized SeNPs (Kora 2018; Ullah et al. 2021; Wang et al. 2024). For instance, Kora (2018) reported spherical SeNPs synthesized by *Bacillus* species with sizes ranging from 50 to 150 nm, while Srivastava and Mukhopadhyay (2015) observed particle sizes between 40 and 120 nm. Similarly, Cinar-Acar (2025) described biogenic SeNPs with sizes extending from 83 to 180 nm. In comparison, the SeNPs obtained in the present study (80–200 nm) fall within this reported range but exhibit moderate aggregation, which may be attributed to differences in biomolecular capping and the synthesis environment. Moreover, compared to previously reported systems, the observed morphology and size distribution suggest that the halotolerant marine strain used in this study is capable of producing SeNPs with comparable structural characteristics. Although the isolate tolerated a range of salinity conditions, the direct influence of salinity on SeNPs yield and physicochemical properties was not specifically investigated in the present study. Overall, the SeNPs obtained in this study are comparable to previously reported systems, with minor variations likely attributable to differences in synthesis conditions.

EDX analysis confirmed selenium as the primary elemental component, with signals of C, O, and N reflecting the closely associated organic matrix. These findings collectively confirm the successful formation of biogenic SeNPs surrounded by stabilizing biomolecules. The colloidal stability of the nanoparticles was further supported by zeta potential analysis, which revealed a strongly negative surface charge (−38 mV). Zeta potential values greater than ± 30 mV are generally considered indicative of good electrostatic stability (Clogston and Patri 2010). The negative charge likely originates from ionized carboxyl, hydroxyl, and other functional groups present in the capping biomolecules (Bulgarini et al. 2021). This value is consistent with previous reports for biogenic SeNPs, including those synthesized by *B. halotolerans* (−37.53 mV) (Ali et al. 2024) and phycosynthesized SeNPs from *Nannochloropsis oceanica* CASA CC20 (−38.5 mV) (Vasanthakumar et al. 2024). Finally, the broad and low-intensity diffraction pattern observed in the XRD analysis confirms the amorphous nature of the synthesized SeNPs. This indicates an absence of long-range crystalline order, which is typical of biologically synthesized SeNPs (Kora 2018; Shi et al. 2021; Spyridopoulou et al. 2021; Cinar-Acar 2025). Amorphous selenium is known to consist of disordered polymeric chains and typically exhibits a characteristic orange–red to brick-red coloration (Sarkar et al. 2011; Kora 2018; Lashani et al. 2024; Cinar-Acar 2025). This amorphous structure may contribute to enhanced biological activity due to increased surface reactivity. In contrast, Miller indices (hkl) apply only to periodic crystal lattices, as they describe specific atomic planes within long-range ordered structures; therefore, they cannot be meaningfully assigned to amorphous materials, which lack translational periodicity (Salem et al. 2023).

The GC–MS results provide a comprehensive chemical basis for understanding the biogenic synthesis of SeNPs by the bacterial filtrate. The proposed mechanism involves three distinct stages: reduction, nucleation, and stabilization. Initially, the reduction of selenite ions to elemental selenium (Se^0^) is likely mediated by the high concentration of bioactive metabolites identified in the extract. Specifically, ethyl iso-allocholate (15.74%) and vitamin E derivatives, which are rich in hydroxyl and carbonyl groups as confirmed by FTIR, act as primary electron donors (reducing agents). Following reduction, the formation of stable SeNPs is facilitated by the presence of 9,12-octadecadienoic acid (11.34%) and 1-heptatriacotanol (8.19%), which serve as capping agents. These fatty acids and long-chain alcohols form an organic layer around the selenium nuclei through hydrophobic interactions, preventing nanoparticle agglomeration and ensuring long-term stability. Furthermore, the presence of benzaldehyde derivatives and polyunsaturated fatty acids in the capping layer likely enhances the observed antimicrobial activity by facilitating the interaction between the SeNPs and the microbial cell membranes. This mechanistic synergy between the identified metabolites explains the dual role of the bacterial filtrate as both a biofactory for nanoparticle synthesis and a source of potent antimicrobial agents.

In addition to their efficient biosynthesis and physicochemical stability, the produced SeNPs exhibited strong broad-spectrum antimicrobial activity against both Gram-positive and Gram-negative bacteria as well as *C. albicans*. This activity is closely related to their structural and surface characteristics. Nanoparticle size is a key determinant of antimicrobial performance (Naga et al. 2025b). The observed size range (80–200 nm) facilitates effective interaction with microbial cell surfaces. Within this range, the relatively smaller nanoparticles may exhibit higher reactivity owing to their larger surface area-to-volume ratio, contributing significantly to the observed antimicrobial activity (Ramamurthy et al. 2013; Kora 2018). The spherical morphology observed by TEM and SEM further enhances surface contact with microbial membranes, promoting adhesion and potential membrane disruption. Additionally, the highly negative zeta potential (−38 mV) contributes to colloidal stability, ensuring uniform dispersion of the SeNPs and increasing the probability of sustained interaction with microbial cells. Despite the negative charge of the bacterial surface, interactions may still occur via hydrophobic interactions, van der Waals forces, and protein-mediated binding mechanisms. Moreover, FTIR analysis indicated that the nanoparticles are coated with biomolecular layers (proteins and polysaccharides), which act as capping agents and enhance biocompatibility, stability, and controlled interaction with microbial membranes (Fritea et al. 2017; Tugarova et al. 2018). The amorphous nature of the SeNPs observed by XRD may also enhance antimicrobial efficacy, due to increased surface reactivity compared to crystalline forms (Shi et al. 2021). Such reactivity may facilitate ROS generation, interaction with thiol-containing enzymes, and disruption of essential cellular processes. EDX analysis revealed a relatively low selenium content (~16%), indicating that the elemental selenium is embedded within or coated by a substantial organic matrix composed of extracellular biomacromolecules. This confirms that the nanoparticles are not bare elemental selenium but are stabilized by extracellular polymeric substances (EPS) (Cremonini et al. 2016). This interpretation is further supported by FTIR spectra, which exhibited prominent absorption bands corresponding to hydroxyl (O–H), amide (C=O), and C–O–C functional groups, indicating the presence of polysaccharides, proteins, and other EPS-related constituents on the nanoparticle surface. Moreover, the XRD pattern showed a broad, diffuse halo without distinct Bragg reflections, confirming the amorphous nature of the SeNPs and suggesting the absence of long-range crystalline order, which is commonly observed in biogenic nanoparticles stabilized by organic coatings that effectively prevent aggregation (Shi et al. 2021). Previous studies have also reported that SeNPs induce oxidative stress, compromise membrane integrity, and inhibit DNA replication in microbial cells (Sarkar et al. 2011; Kora 2018). Although the specific antimicrobial mechanism of the SeNPs was not experimentally validated in the present study, the observed activity can be reasonably interpreted based on previously established pathways, including the disruption of microbial cell membranes, the generation of ROS, and interaction with intracellular biomolecules (Chan et al. 2025). This limitation has been acknowledged and should be addressed in future studies. Functionally, the biosynthesized SeNPs demonstrated differential antimicrobial efficacy across the tested microorganisms. The MBC/MIC and MFC/MIC ratios were used to evaluate whether the effect was bactericidal/fungicidal or bacteriostatic/fungistatic, with ratios ≤ 4 indicating killing activity and > 4 indicating growth inhibition (Ishak et al. 2025). Among the tested microorganisms, *S. aureus* exhibited the highest susceptibility, which aligns with its large inhibition zone and statistical significance. This enhanced sensitivity may be attributed to the structural characteristics of Gram-positive bacteria, which lack an outer membrane and possess a porous, thick peptidoglycan layer that facilitates nanoparticle interaction with the plasma membrane. In contrast, Gram-negative bacteria such as *P. aeruginosa* exhibited relatively lower susceptibility due to the presence of an outer membrane that acts as a permeability barrier, supplemented by intrinsic resistance mechanisms such as efflux pumps. For the fungal strain *C. albicans*, antifungal activity was assessed using MFC determination. The MFC/MIC ratio of 4 indicates a fungistatic rather than fungicidal effect, which may be attributed to the complex and dense chitin/glucan structure of fungal cell walls that limits nanoparticle penetration. Overall, the antimicrobial performance of the SeNPs appears to be driven by a combination of physicochemical properties and biological interactions, primarily involving ROS generation and membrane disruption. This strong bactericidal effect compared to the predominantly fungistatic response highlights the influence of microbial cell wall architecture on nanoparticle susceptibility. A direct comparison with the standard antibiotic gentamicin demonstrated that the synthesized SeNPs exhibited comparable inhibition zones against several tested strains, highlighting their potential as alternative antimicrobial agents. However, variations in susceptibility were observed depending on the microbial species, reflecting differences in cell wall structure and intrinsic resistance mechanisms. These findings are consistent with previously reported studies, further supporting the antimicrobial efficacy of biogenic SeNPs (Table 4).

**Table 4:** Comparison of antimicrobial activity of biogenic SeNPs with previously reported studies

Tested microorganism	Inhibition zone (mm)	SeNP source	Reference
*E. coli*	3.7 ± 0.6	*Ralstonia insidiosa*	(Ahmed et al. 2026)
	14.12 ± 0.89	*B. paramycoides*	(Liu et al. 2023)
	11.3 ± 0.58	*B. subtilis* BSN313	(Ullah et al. 2021)
	0.30 ± 0.01	*Kluyveromyces marxianus*	(Cinar-Acar 2025)
	17	*Bacillus* sp.	(Greeshma and Mahesh 2019)
*S. epidermidis*	4.7 ± 0.7	*R. insidiosa*	(Ahmed et al. 2026)
*S. aureus*	16.01 ± 0.51	*B. paramycoides*	(Liu et al. 2023)
	13	*Bacillus* sp.	(Greeshma and Mahesh 2019)
	15 ± 1.0	*B. subtilis* BSN313	Ullah et al. 2021
	No zone	*K. marxianus*	(Cinar-Acar 2025)
*P. aeruginosa*	12 ± 1.0	*B. subtilis* BSN313	(Ullah et al. 2021)
	15	*Bacillus* sp.	(Greeshma and Mahesh 2019)
	5.7 ± 0.6	*R. insidiosa*	(Ahmed et al. 2026)
	1.2 ± 0.02	*K. marxianus*	(Cinar-Acar 2025)
*K. pneumoniae*	2.7 ± 0.6	*R. insidiosa*	(Ahmed et al. 2026)

## Limitations and future perspectives

The present study has some limitations, including the lack of experimental validation of the antimicrobial mechanism, the absence of comparative analysis with chemically synthesized SeNPs, and the limited investigation of salinity effects on nanoparticle properties. In addition, cytotoxicity and biocompatibility assessments were not performed. Future studies should focus on mechanistic investigations (e.g., ROS generation and membrane damage), optimization of synthesis conditions, evaluation of biocompatibility, and assessment of large-scale production feasibility.

## Conclusions

This study reports the successful isolation of a halotolerant marine *B. sonorensis* 2MNHR strain capable of efficiently reducing sodium selenite and mediating the extracellular production of SeNPs. Only one of the tested isolates consistently reduced selenite under aerobic conditions, and extracellular activity was validated using cell-free supernatant assays. Biosynthesized SeNPs were characterized as spherical, amorphous, and nanoscale, with remarkable colloidal stability (− 38 mV). Spectroscopic and microscopic investigations confirmed the effective bioreduction of selenite to elemental selenium and demonstrated the critical role of bacterial biomolecules in nanoparticle stability. Furthermore, the biogenic SeNPs demonstrated substantial antibacterial and antifungal activity against both Gram-positive and Gram-negative bacteria, as well as *C. albicans*, indicating their potential biomedical applications. The primary objectives of this investigation were successfully achieved, and despite the noted limitations, the overall findings provide strong evidence that halotolerant marine bacteria represent promising, sustainable, and eco-friendly platforms for the extracellular production of stable SeNPs with significant antimicrobial potential.

## Data Availability

No datasets were generated or analysed during the current study.
